# Maternal Exposure to Particulate Air Pollution and Term Birth Weight: A Multi-Country Evaluation of Effect and Heterogeneity

**DOI:** 10.1289/ehp.1205575

**Published:** 2013-02-06

**Authors:** Payam Dadvand, Jennifer Parker, Michelle L. Bell, Matteo Bonzini, Michael Brauer, Lyndsey A. Darrow, Ulrike Gehring, Svetlana V. Glinianaia, Nelson Gouveia, Eun-hee Ha, Jong Han Leem, Edith H. van den Hooven, Bin Jalaludin, Bill M. Jesdale, Johanna Lepeule, Rachel Morello-Frosch, Geoffrey G. Morgan, Angela Cecilia Pesatori, Frank H. Pierik, Tanja Pless-Mulloli, David Q. Rich, Sheela Sathyanarayana, Juhee Seo, Rémy Slama, Matthew Strickland, Lillian Tamburic, Daniel Wartenberg, Mark J. Nieuwenhuijsen, Tracey J. Woodruff

**Affiliations:** 1 Centre for Research in Environmental Epidemiology (CREAL), Barcelona, Spain; 2 Municipal Institute of Medical Research (IMIM-Hospital del Mar), Barcelona, Spain; 3 CIBER Epidemiologia y Salud Pública (CIBERESP), Spain; 4 National Center for Health Statistics, Centers for Disease Control and Prevention, Hyattsville, Maryland, USA; 5 School of Forestry and Environmental Studies, Yale University, New Haven, Connecticut, USA; 6 Department of Clinical and Experimental Medicine, University of Insubria, Varese, Italy; 7 University of British Columbia, School of Population and Public Health, Vancouver, British Columbia, Canada; 8 Department of Environmental Health, Emory University, Atlanta, Georgia, USA; 9 Institute for Risk Assessment Sciences, Utrecht University, Utrecht, the Netherlands; 10 Institute of Health & Society, Newcastle University, Newcastle upon Tyne, United Kingdom; 11 Department of Preventive Medicine, School of Medicine of the University of São Paulo, São Paulo, Brazil; 12 Department of Preventive Medicine, Ewha Womans University, Seoul, Republic of Korea; 13 Department of Occupational and Environmental Medicine, Inha University, Incheon, Republic of Korea; 14 Generation R Study Group, Erasmus Medical Center, Rotterdam, the Netherlands; 15 Urban Environment and Safety, TNO, Utrecht, the Netherlands; 16 Centre for Research, Evidence Management and Surveillance, Sydney, Australia; 17 South Western Sydney Local Health Districts, Sydney, Australia; 18 School of Public Health and Community Medicine, University of New South Wales, Sydney, Australia; 19 Department of Environmental Science, Policy, and Management, University of California–Berkeley, Berkeley, California, USA; 20 Department of Environmental Health, Harvard School of Public Health, Boston, Massachusetts, USA; 21 Team of Environmental Epidemiology Applied to Reproduction and Respiratory Health, INSERM, and; 22 Grenoble University, U823, Institut Albert Bonniot, Grenoble, France; 23 School of Public Health, University of California–Berkeley, Berkeley, California, USA; 24 North Coast Area Health Service, Lismore, New South Wales, Australia; 25 University Centre for Rural Health–North Coast, University of Sydney, Sydney, Australia; 26 Department of Occupational and Environmental Health, Università di Milano, Milan, Italy; 27 Department of Public Health Sciences, University of Rochester School of Medicine and Dentistry, Rochester, New York, USA; 28 Seattle Children’s Research Institute, University of Washington, Seattle, Washington, USA; 29 Centre for Health Services and Policy Research, University of British Columbia, Vancouver, Canada; 30 UMDNJ–Robert Wood Johnson Medical School, Piscataway, New Jersey, USA; 31 Center for Reproductive Health and the Environment, University of California–San Francisco, San Francisco, California, USA

**Keywords:** air pollution, fetal growth, heterogeneity, ICAPPO, low birth weight, meta-analysis, meta-regression, multi-center study, particulate matter, pregnancy

## Abstract

Background: A growing body of evidence has associated maternal exposure to air pollution with adverse effects on fetal growth; however, the existing literature is inconsistent.

Objectives: We aimed to quantify the association between maternal exposure to particulate air pollution and term birth weight and low birth weight (LBW) across 14 centers from 9 countries, and to explore the influence of site characteristics and exposure assessment methods on between-center heterogeneity in this association.

Methods: Using a common analytical protocol, International Collaboration on Air Pollution and Pregnancy Outcomes (ICAPPO) centers generated effect estimates for term LBW and continuous birth weight associated with PM_10_ and PM_2.5_ (particulate matter ≤ 10 and 2.5 µm). We used meta-analysis to combine the estimates of effect across centers (~ 3 million births) and used meta-regression to evaluate the influence of center characteristics and exposure assessment methods on between-center heterogeneity in reported effect estimates.

Results: In random-effects meta-analyses, term LBW was positively associated with a 10-μg/m^3^ increase in PM_10_ [odds ratio (OR) = 1.03; 95% CI: 1.01, 1.05] and PM_2.5_ (OR = 1.10; 95% CI: 1.03, 1.18) exposure during the entire pregnancy, adjusted for maternal socioeconomic status. A 10-μg/m^3^ increase in PM_10_ exposure was also negatively associated with term birth weight as a continuous outcome in the fully adjusted random-effects meta-analyses (–8.9 g; 95% CI: –13.2, –4.6 g). Meta-regressions revealed that centers with higher median PM_2.5_ levels and PM_2.5_:PM_10_ ratios, and centers that used a temporal exposure assessment (compared with spatiotemporal), tended to report stronger associations.

Conclusion: Maternal exposure to particulate pollution was associated with LBW at term across study populations. We detected three site characteristics and aspects of exposure assessment methodology that appeared to contribute to the variation in associations reported by centers.

The developing fetus is known to be susceptible to environmental insults (Stillerman et al. 2008). A growing body of evidence has associated maternal exposure to ambient air pollution with a range of adverse pregnancy outcomes including low birth weight (LBW), intra-uterine growth retardation, preterm birth, stillbirth, and congenital anomalies (Glinianaia et al. 2004; Sapkota et al. 2010; Šrám et al. 2005; Vrijheid et al. 2011). However, notable inconsistencies among the findings of these studies (Parker and Woodruff 2008; Parker et al. 2011; Woodruff et al. 2009) have hindered the ability of policy makers to incorporate the research evidence into policy.

Discrepancies among previous studies may reflect genuine differences in the study settings, may be a consequence of specific biases, or may arise from differences in study designs and exposure assessments (Parker and Woodruff 2008; Woodruff et al. 2009). Study setting characteristics that may contribute to variation in reported associations include the demographic characteristics of the study population, the major sources of pollutants, the size distribution of particulate pollutants [e.g., PM_2.5_:PM_10_ ratio (particulate matter ≤ 2.5 and 10 µm)], maternal time–activity patterns, the study period, the degree of confounding by socioeconomic status (SES), and the underlying prevalence of adverse pregnancy outcomes. Relevant study design characteristics include the sources of data for feto-maternal characteristics (e.g., birth certificates, questionnaires, or hospital records), inclusion/exclusion criteria, outcome definitions (e.g., birth weight as a continuous variable, LBW, or small for gestational age), and the analysis of potential confounders and/or effect modifiers. Differences in exposure assessment include applied methods for assessing the exposure (e.g., proximity-based, monitor-based, or model-based methods), exposure time windows, exposure contrasts (e.g., spatial, temporal, or spatiotemporal), the availability of data for specific pollutants, and the analysis of associations with combinations of pollutants (Parker and Woodruff 2008).

The International Collaboration on Air Pollution and Pregnancy Outcomes (ICAPPO) was established to investigate the association between maternal exposure to ambient air pollution and pregnancy outcomes across multiple centers and to understand how differences in study settings and methods contribute to variations in findings (i.e., between-center heterogeneity) (Parker et al. 2011; Woodruff et al. 2009, 2010). To achieve this goal, we previously discussed methodological differences in the published studies (Woodruff et al. 2009), described the collaborative centers (Woodruff et al. 2010), and presented preliminary estimates of effects for each center (Parker et al. 2011).

The overarching aim of this analysis was to evaluate the association between maternal exposure to particulate air pollution and term birth weight and LBW. Toward this aim, we combined effect estimates of the individual ICAPPO centers and assessed the between-center heterogeneity in these associations. The application of a common analysis protocol across ICAPPO centers provided a unique opportunity to separate the contribution of the analytical design to between-center heterogeneity, and enabled us to assess the impact of center characteristics and exposure assessment methods on the variation in reported effect estimates by each center.

## Methods

*Overview.* Our study was based on estimates of effects [odds ratio (OR) for LBW and regression coefficients for birth weight] that were uniformly generated and reported by each ICAPPO center according to a common protocol (Parker et al. 2011). Ambient levels of PM_10_ and PM_2.5_ were used as indicators of particulate air pollution. Our analysis was focused principally on the association between maternal exposure to PM_10_ during the entire pregnancy and term LBW (birth weight < 2500 g at 37–42 completed weeks of gestation) because this was reported by most ICAPPO centers. We also conducted additional analyses of data from subsets of ICAPPO centers that stratified PM_10_–term LBW analyses by the exposure time window (i.e., the first, second, and third trimester), analyzed birth weight as a continuous outcome variable, and estimated the association between maternal exposure to PM_2.5_ and term LBW. We synthesized the effect estimates across the centers by applying a meta-analysis framework. The effects of center characteristics and exposure assessment methods on between-center heterogeneity in effect estimates were explored using a meta-regression framework. The meta-analyses and meta-regressions were conducted using the R statistical package (http://cran.r-project.org/), libraries meta, rmeta, and metafor.

*ICAPPO.* Our analysis relied on effect estimates provided by fourteen ICAPPO centers from nine countries with more than three million singleton term births (Table 1). For the ICAPPO analysis, the centers reanalyzed existing data sets that had been created to evaluate the impacts of maternal exposure to air pollution on pregnancy outcomes. The centers relied on outcome data available from routinely collected administrative records (birth certificates) or data collected for a specific study (Bell et al. 2007, 2008; Brauer et al. 2008; Darrow et al. 2011; Gehring et al. 2011; Glinianaia et al. 2008; Gouveia et al. 2004; Ha et al. 2004; Jalaludin et al. 2007; Lepeule et al. 2010; Mannes et al. 2005; Morello-Frosch et al. 2010; Pesatori et al. 2008; Rich et al. 2009; Slama et al. 2009; van den Hooven et al. 2009). More detailed description of the ICAPPO centers has been previously published (Parker et al. 2011; Woodruff et al. 2010).

**Table 1:** Exposure assessment methodologies and characteristics of the ICAPPO centers.

Center, location	Reference	Study period	Study area (km2)	No. of births	Measure of SES	Term LBW (%)	Median PM10 (μg/m3)	PM10 IQR (μg/m3)	Median PM2.5 (μg/m3)	PM2.5/PM10 ratioa	Exposure assessment	Exposure contrast
Atlanta, GA, USA	Darrow et al. 2011	1996–2004	4,538	325,221	Maternal education	2.62	23.5	3.1	15.8	0.67	Monitor	Temporal
California, USA	Morello-Frosch et al. 2010	1996–2006	423,970	1,714,509	Maternal education	2.43	28.9	16.1	16.5	0.57	Monitor	Spatiotemporal
Connecticut and Massachusetts, USA	Bell et al. 2007, 2008	1999–2002	41,692	173,042	Maternal education	2.16	22	7.4	20	0.91	Monitor	Spatiotemporal
EDEN, Poitiers and Nancy, France	Lepeule et al. 2010	2003–2006	480	1233	Age at completion of education	2.11	19	3	—	—	Monitor	Spatiotemporal
Lombardy, Italy	Pesatori et al. 2008	2004–2006	23,865	213,542	Maternal education	2.71	49	10	—	—	Monitor	Spatiotemporal
PAMPER, Newcastle upon Tyne, UK	Glinianaia et al. 2008	1962–1992	63	81,953	Area-level indicatorb	3.19	32.8c	87.8c	—	—	Model	Spatiotemporal
New Jersey, USA	Rich et al. 2009	1999–2003	22,592	87,281	Maternal education	2.75	28	6.9	13.7	0.49	Monitor	Spatiotemporal
PIAMA, North, West, and Center of the Netherlands	Gehring et al. 2011	1996–1997	12,000	3,471	Maternal education	1.15	40.5	6.7	20.3	0.50	Model	Spatiotemporal
Generation R, Rotterdam, Netherlands	van den Hooven et al. 2009	2002–2006	150	7,296	Maternal education	2.26	32.8	1.1	—	—	Model	Spatial
São Paulo, Brazil	Gouveia et al. 2004	2005	1,500	158,791	Maternal education	3.77	40.3	2.9	—	—	Monitor	Temporal
Seattle, WA, USA	Sathyanarayana S, Karr C, unpublished data	1998–2005	17,800	301,880	Maternal education	1.56		—	10.2	—	Monitor	Spatiotemporal
Seoul, South Korea	Ha et al. 2004	1998–2000	605	372,319	Maternal education	1.45	66.5	10.9	—	—	Monitor	Temporal
Sydney, Australia	Jalaludin et al. 2007	1998–2004	12,145	279,015	Area-level indicatord	1.62	16.5	8.2	—	—	Monitor	Temporal
Vancouver, BC, Canada	Brauer et al. 2008	1999–2002	3,300	66,467	Area-level indicatore	1.35	12.5	1.4	3.98	0.32	Monitor	Spatiotemporal
aRatio of PM10 and PM2.5 median levels. bThe Townsend Deprivation Score is an area-based measure of material deprivation calculated for each enumeration district (~ 200 households) based on 1971, 1981, and 1991 census data. cBlack smoke (~ PM4) was used as a measure of particulate air pollution. dThe Australian Bureau of Statistics (2003) Index of Relative Socio-economic Disadvantage uses a range of census factors and is assigned to each census collection district (~ 200 households). eThe percentage of women with postsecondary education.												

Participating centers were initially asked to provide information on their study location and period, available air pollutants, number of births, prevalence of term LBW, exposure assessment method, and available covariate data (Parker et al. 2011; Woodruff et al. 2010). Based on this information, ICAPPO participants developed a common analytical protocol detailing the inclusion criteria, outcomes and covariates of interest, statistical models, and sensitivity analyses. This protocol also specified a standardized way of reporting the results (Parker et al. 2011). Each center was asked to reanalyze its existing data set according to this protocol. The analyses were limited to live-born, singleton, term births with known birth weight, maternal education (or another measure of SES), dates of birth and conception, and ambient PM_10_ or PM_2.5_ concentrations during the entire pregnancy.

*Primary meta-analysis.* According to the common protocol, ICAPPO centers initially estimated the association between term LBW and maternal exposure to PM_10_ averaged over the entire pregnancy. Each center constructed three logistic regression models to estimate the odds of term LBW associated with each 10-μg/m^3^ increase in average PM_10_ exposure levels during the entire pregnancy: *a*) without any adjustment, *b*) with adjustment only for maternal SES, and *c*) with adjustment for maternal SES and center-specific covariates (e.g., maternal age, maternal ethnicity, maternal smoking, parity, and infant sex). For these center-specific covariates, there was no recommendation in the ICAPPO protocol and the centers had the flexibility to independently choose the suitable ones according to their settings. This selection of extra covariates used by each center has been reported elsewhere (Parker et al. 2011). The construction of these three models was to evaluate the effect of adjustment for the maternal SES and other covariates on the combined effect estimates.

ICAPPO participants chose maternal education as a common indicator of maternal SES (Parker et al. 2011). If maternal education data were not available, area-level measures of SES were used [for the PAMPER (Particulate Matter and Perinatal Events Research) study: Townsend Deprivation Score; for the Sydney study: Index of Relative Socioeconomic Disadvantage; and for the Vancouver study: percentage of women with postsecondary education]. The PAMPER study (Newcastle upon Tyne, UK) provided exposure data only for black smoke, which approximates particulate matter with an aerodynamic diameter ≤ 4 µm (PM_4_) and has been shown to be a reasonable surrogate for PM_10_ (Muir and Laxen 1995). The estimates of effect for the PAMPER study were therefore analyzed alongside the studies with PM_10_ measures.

We used meta-analysis to estimate combined ORs across the centers. Between-center heterogeneity was quantified using the between-center variance of effect estimates, τ^2^. The statistical significance of between-center heterogeneity was tested by Cochran’s Q test. The *I*^2^ statistic with 95% confidence intervals (CI) was used to estimate the proportion of total variation in effect estimates across centers that was attributable to the between-center heterogeneity (τ^2^) rather than within-center error (Higgins 2008). If there was significant between-center heterogeneity (i.e., Cochran’s Q test *p*-value < 0.05), DerSimonian–Laird random-effects models were used for meta-analysis (DerSimonian and Laird 1986); otherwise, fixed-effects models were conducted using the Mantel–Haenszel method (Mantel and Haenszel 1959). Associations with a Cochran’s Q test *p*-value between 0.05 and 0.10 were estimated using both fixed- and random-effects models (Higgins et al. 2002).

*Additional meta-analyses.* Exposure window period. Nine centers analyzed the association between maternal exposure to PM_10_ and term LBW stratified by the trimester of exposure and adjusted for maternal SES. We carried out meta-analyses using these reported stratified ORs to evaluate the impact of the exposure window period on the association between PM_10_ exposure and term LBW.

Birth weight as a continuous variable. A subset of 11 centers (all centers but Connecticut and Massachusetts, New Jersey, and Seattle, Washington) estimated the change in term birth weight as a continuous outcome variable (grams) associated with each 10-μg/m^3^ increase in PM_10_ exposure levels averaged over the entire pregnancy using three sets of linear regression models with predictors as described for the primary analysis. The combined effect estimates across the centers were calculated using meta-analysis, as described above.

PM_2.5_ exposure. Seven centers had data on PM_2.5_ (Table 1) and reported the odds of term LBW associated with each 10-μg/m^3^ increase in average PM_2.5_ exposure levels during the entire pregnancy, using three sets of logistic regression models as described for the primary analysis. We synthesized the effect estimates across these centers using meta-analysis as described before.

*Sensitivity analyses.* We checked the robustness of all meta-analyses results to the omission of influential centers that were identified using DFBETAS, which indicates the change in estimated coefficients after excluding a center from the meta-analysis (Viechtbauer and Cheung 2010). Centers with an absolute DFBETAS value > 1 (i.e., that resulted in ≥ 1-SD change in the estimated coefficient when omitted) were considered influential (Viechtbauer and Cheung 2010).

*Meta-regressions.* To assess between-center heterogeneity, we hypothesized that the following characteristics of the centers could have an impact on the estimated risk by each center: study area (square kilometers), length of the study (years), number of births, continent of the study location, latitude of the study location (degrees), percentage of term LBW births, median PM_10_ exposure levels (micrograms per cubic meter), median PM_2.5_ exposure levels (micrograms per cubic meter), interquartile range of PM_10_ exposure levels (micrograms per cubic meter), and PM_2.5_:PM_10_ ratio. In addition, we hypothesized that the use of model-based versus monitor-based exposure assessment, and temporal versus spatiotemporal exposure contrasts could influence center-specific estimates. Temporal exposure contrasts account for temporal variation in pollutant levels by assigning measurements from a single monitoring station to all study subjects, whereas spatiotemporal contrasts account for both spatial and temporal components of variation in air pollution levels when estimating exposure.

For associations between maternal PM_10_ exposure during the entire pregnancy and term LBW that showed between-center heterogeneity, we performed separate univariate meta-regressions using center-specific log-transformed ORs as outcome and each of the center or exposure assessment characteristics listed above as predictors. In effect, these meta-regressions quantified the potential impact of these factors on the estimates of effects (ORs) reported by each center. We excluded the Generation R study (Table 1) from the analysis comparing temporal and spatiotemporal exposure contrasts because its exposure assessment was based on a dispersion model, which is essentially a spatial approach without any temporal adjustment. Analyses of the influence of PM_2.5_ and PM_2.5_:PM_10_ ratios were based on the seven centers with PM_2.5_ data.

## Results

*Characteristics of ICAPPO centers.* Of the 14 ICAPPO centers included in our analyses (Table 1), 6 were North American, 5 European, 1 South American, 1 Asian, and 1 was Oceanian. Our analysis included > 3 million births (ranging from a little more than 1,000 to almost 2 million) generally occurring between late 1990s and mid-2000s.

**Figure 1:**
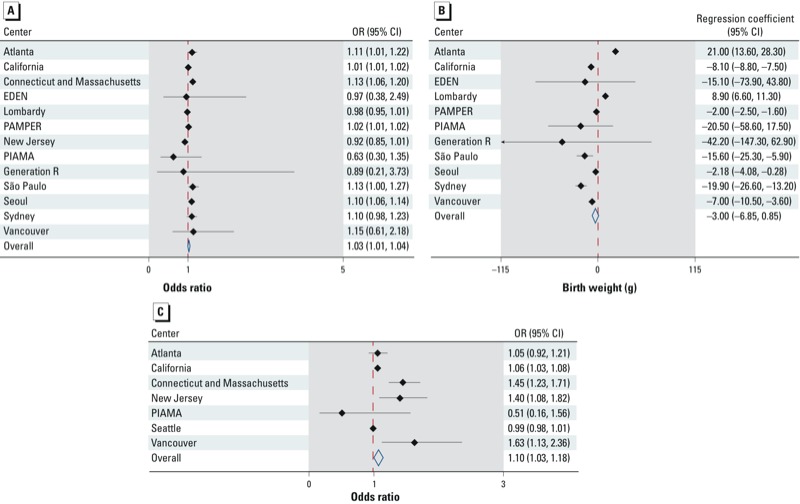
Forest plots for the random-effects meta-analysis of the SES-adjusted OR (95% CI) for the associations between term LBW and PM_10_ exposure during the entire pregnancy (*A*), term birth weight and PM_10_ exposure during the entire pregnancy (*B*), and between term LBW and PM_2.5_ exposure during the entire pregnancy (*C*).

*Primary meta-analysis.* Thirteen centers provided estimates for the association between maternal exposure to PM_10_ and term LBW [Figure 1A; see also Supplemental Material, Figure S1, for forest plots (http://dx.doi.org/10.1289/ehp.1205575)]. There was statistically significant (Cochran’s Q test *p*-value < 0.05) between-center heterogeneity in effect estimates (ORs) reported by these centers, with τ^2^ ranging between 0.0003 and 0.0004 (Table 2). Therefore we used random-effects models to estimate combined ORs across the centers, which indicated a positive association between term LBW and average maternal exposure to PM_10_ during the entire pregnancy before adjustment (OR = 1.04; 95% CI: 1.01, 1.06) and after adjustment for SES (OR = 1.03; 95% CI: 1.01, 1.05) and SES plus center-specific covariates (OR = 1.02; 95% CI: 1.01, 1.04) (Table 2).

**Table 2:** Combined random-effects ORs (95% CIs) for term LBW in association with a 10-μg/m^3^ increase in average maternal exposure to PM_10_ and PM_2.5_ during pregnancy, and corresponding indicators of between-center heterogeneity across ICAPPO centers.

Meta-analysis	Combined estimate		Heterogeneity		
	OR (95% CI)	p-Value	τ2	p-Valuea	I2 (95% CI)
PM10 (13 centers)
Unadjusted	1.04 (1.01, 1.06)	< 0.01	0.0004	< 0.01	76.5% (59.9%, 86.2%)
Adjusted for maternal SES	1.03 (1.01, 1.05)	< 0.01	0.0003	< 0.01	79.4% (65.4%, 87.7%)
Adjusted for maternal SES and center-specific covariates	1.02 (1.01, 1.04)	0.01	0.0003	0.01	54.3% (14.5%, 75.6%)
PM2.5 (7 centers)b
Unadjusted	1.17 (1.08, 1.26)	< 0.01	0.0055	< 0.01	92.3% (86.7%, 95.6%)
Adjusted for maternal SES	1.10 (1.03, 1.18)	< 0.01	0.0039	< 0.01	89.7% (81.3%, 94.3%)
Adjusted for maternal SES and center-specific covariates	1.04 (0.99, 1.09)	0.09	0.0013	< 0.01	68.5% (30.4%, 85.7%)
ap-Value for Cochran’s Q test for heterogeneity. bIncluding Atlanta, California, Connecticut and Massachusetts, New Jersey, PIAMA, Seattle, and Vancouver.					

*Additional meta-analyses.* Exposure window period. Nine centers reported estimates for the association between PM_10_ and term LBW stratified by the trimester of exposure and adjusted for maternal SES [see Supplemental Material, Figure S2, for forest plots (http://dx.doi.org/10.1289/ehp.1205575)]. All combined trimester-specific ORs for term LBW in association with a 10-μg/m^3^ increase in PM_10_ were positive and comparable in magnitude based on fixed-effects models adjusted for maternal SES, with ORs of 1.01 (95% CI: 1.01, 1.01), 1.01 (95% CI: 1.01, 1.02), and 1.02 (95% CI: 1.01, 1.02) for the first, second, and third trimesters, respectively (Table 3). Corresponding random-effects ORs were smaller, with a combined OR for the first trimester of 1.0 (95% CI: 1.00, 1.01) (Cochran’s Q test *p* = 0.06; *I*^2^ = 45.8%; 95% CI: 0, 74.9%) (Table 3).

**Table 3:** Combined adjusteda ORs (95% CIs) of term LBW in association with a 10-μg/m^3^ increase in average of PM_10_ exposure levels during each trimester of exposure.^b^

Trimester of exposure	Combined risk		Heterogeneity	
	OR (95% CI)	p-Value	I2 (95% CI)	p-Valuec
1st trimester (fixed-effects model)	1.01 (1.01, 1.01)	< 0.001	45.8% (0.0%, 74.9%)	0.064
1st trimester (random-effects model)	1.00 (1.00, 1.01)	0.325
2nd trimester (fixed-effect smodel)	1.01 (1.01, 1.02)	< 0.001	25.7% (0.0%, 65.2%)	0.213
2nd trimester (random-effects model)	1.01 (1.01, 1.02)	< 0.001
3rd trimester (fixed-effects model)	1.02 (1.01, 1.02)	< 0.001	42.7% (0.0%, 73.6%)	0.075
3rd trimester (random-effects model)	1.01 (1.00, 1.02)	0.001
aAdjusted for maternal SES. bORs from nine centers were included in the meta-analysis. cp‑Value for Cochran’s Q test for heterogeneity.				

Birth weight as a continuous variable. Eleven centers estimated the change in term birth weight as a continuous outcome variable (grams) associated with each 10-μg/m^3^ increase in PM_10_ exposure levels [Figure 1B; see also Supplemental Material, Figure S3, for forest plots (http://dx.doi.org/10.1289/ehp.1205575)]. The Cochran’s Q test of heterogeneity was significant (*p* < 0.05) for all meta-analyses of PM_10_ and birth weight (data not shown). Random-effects meta-analyses indicated a negative association between term birth weight and a 10-μg/m^3^ increase in PM_10_, with unadjusted, SES-adjusted, and SES- plus center-specific covariate–adjusted estimated decreases of –2.7 g (95% CI: –7.2, 1.7 g, *p* = 0.23), –3.0 g (95% CI: –6.9, 0.9 g, *p* = 0.13), and –8.9 g (95% CI: –13.2, –4.6 g, *p* < 0.01), respectively.

PM_2.5_ exposure. Seven centers reported the odds of term LBW associated with each 10-μg/m^3^ increase in PM_2.5_ exposure levels [Figure 1C; see also Supplemental Material, Figure S4, for forest plots (http://dx.doi.org/10.1289/ehp.1205575)]. All meta-analyses showed statistically significant between-study heterogeneity (Table 2). The random-effects meta-analyses demonstrated positive associations with term LBW, with statistically significant ORs based on unadjusted and SES-adjusted models, but not the model adjusted for maternal SES and center-specific covariates (Table 2).

*Sensitivity analyses.* The studies that were most frequently classified as influential were the California study (the largest center with more than 1.7 million births), the PAMPER study (covering a long time period with wide variation in sources and levels of exposure to black smoke, which was used as a surrogate for PM_10_), and the Lombardy study [a relatively large study of a heterogeneous region including a metropolitan area (Milan), a large mainly agricultural area (Po valley), and a northern mountainous area] [see Supplemental Material, Table S1 (http://dx.doi.org/10.1289/ehp.1205575)]. The meta-analyses were generally robust to the exclusion of influential studies with regard to the magnitude of the estimated associations (data not shown), but there were some exceptions. For example, the unadjusted OR for LBW with a 10-μg/m^3^ increase in PM_2.5_ increased from 1.17 (95% CI: 1.08, 1.26) to 1.20 (95% CI: 1.00, 1.45) after California was excluded, and decreased to 1.06 (95% CI: 0.99, 1.13) after exclusion of Connecticut and Massachusetts. In addition, the average estimated reduction in SES-adjusted term birth weight with a10-μg/m^3^ increase in PM_10_ (random-effects model) increased from –3.0 g (95% CI: –6.9, 0.9 g) based on all centers (Figure 1B) to –5.5 g (95% CI: –9.3, –1.6 g) after excluding Atlanta, and to –4.9 g (95% CI: –8.6, –1.1 g) after excluding Lombardy. Similarly, the reduction in unadjusted mean birth weight increased from –2.7 g (95% CI: –7.2, 1.7 g) based on all centers (see Supplemental Material, Figure S3A) to –6.6 g (95% CI: –11.7, –1.5 g) and –5.0 g (95% CI: –10.0, 0.0 g) after removing Atlanta and Lombardy, respectively.

*Meta-regressions.* Of the characteristics we evaluated, median PM_2.5_ exposure levels, PM_2.5_:PM_10_ ratio, and exposure contrast (temporal vs. spatiotemporal approach) influenced the between-center heterogeneity in the reported PM_10_-term LBW associations, with centers that had higher median PM_2.5_ exposure levels and PM_2.5_:PM_10_ ratios, and that used a temporal exposure contrast, reporting stronger associations in most cases (Table 4). The results of leave-one-out sensitivity analyses for these three meta-regressions were generally consistent with those of meta-regressions including all centers (data not shown). However, removing Connecticut and Massachusetts almost doubled the meta-regression coefficient for the PM_2.5_:PM_10_ ratio and nullified the association for median PM_2.5_ exposure levels, and the meta-regression estimate for median PM_2.5_ exposure levels was null after New Jersey was excluded.

**Table 4:** Meta-regression coefficients (95% CIs) from separate models estimating the effect of a 1-μg/m^3^ increase in the center median PM_2.5_ level, a 100% increase in PM_2.5_:PM_10_ ratio, or the use of a temporal versus spatiotemporal exposure contrast on SES-adjusted center-specific log-ORs for the association between a 10-μg/m^3^ increase in mean PM_10_ during pregnancy and term LBW.

Meta-regression	Temporal vs. spatiotemporal approacha,b		Median PM2.5 levels		PM2.5:PM10 ratio	
	Regression coefficient (95% CI)	Residual heterogeneity (τ2) (95% CI)	Regression coefficient (95% CI)	Residual heterogeneity (τ2) (95% CI)	Regression coefficient (95% CI)	Residual heterogeneity (τ2) (95% CI)
Unadjusted	0.07 (0.03, 0.11)	0.0033 (0.0007, 0.0259)*	0.03 (0.01, 0.05)	0.0105 (0.0, 0.3901)	0.47 (0.13, 0.82)	0.0019 (0.0, 0.3808)
Adjusted for maternal SES	0.08 (0.05, 0.12)	0.0015 (0.0001, 0.0116)*	0.02 (0.00, 0.04)	0.0077 (0.0, 0.3405)	0.39 (0.15, 0.64)	0.0010 (0.0, 0.3776)
Adjusted for maternal SES and center-specific covariates	0.06 (0.02, 0.10)	0.0006 (0.0, 0.0079)	0.01 (0.00, 0.03)	0.0 (0.0, 0.0608)	0.19 (0.01, 0.37)	0.0 (0.0, 0.1421)
aRegression coefficients for using temporal approach compared with the spatiotemporal approach. bGeneration R cohort was excluded because its exposure assessment was based on a dispersion model which is a spatial approach. *Cochran’s Q test p < 0.05.						

Meta-regressions for the exposure contrast showed statistically significant heterogeneity in residuals (from both unadjusted and SES-adjusted models), whereas meta-regressions of the median PM_2.5_ exposure levels and PM_2.5_:PM_10_ ratios did not (Table 4). Heterogeneity was reduced when ORs were adjusted for center-specific selection of covariates in addition to SES. Associations between center-specific log-ORs and other center or study characteristics were not statistically significant (data not shown).

## Discussion

To our knowledge, this study is the largest multicenter study so far reporting on the association between air pollution and fetal growth using a common analytical protocol. We synthesized reported estimates of effects by 14 ICAPPO centers around the globe and for the first time quantified the impacts of study settings and aspects of applied exposure assessment methods on between-center heterogeneity in the reported effect estimates (ORs) for this association. We found that maternal exposures to PM_10_ and PM_2.5_ during the entire pregnancy were positively associated with term LBW. For PM_10_, all trimester-specific exposures were associated with slightly increased odds of term LBW. Furthermore, term birth weight was reduced in association with average PM_10_ exposure over the entire pregnancy. Most associations showed between-center heterogeneity in the center-specific estimates of associations. Meta-regression assessments of factors possibly affecting PM_10_-term LBW associations indicated that heterogeneity was influenced by median PM_2.5_ exposure levels, the PM_2.5_:PM_10_ ratio, and the applied exposure contrast (temporal vs. spatiotemporal).

*Primary meta-analyses.* Making decisions about the risks from environmental exposure in the policy or clinical setting requires synthesizing and interpreting the available epidemiologic evidence, which may include evaluating a number of relevant studies with different designs. A recent systematic review and meta-analysis of the available evidence on the association between particulate pollution and pregnancy outcomes reported by Sapkota et al. (2010) estimated a combined OR of 1.02 (95% CI: 0.99, 1.05) for LBW in association with a 10-μg/m^3^ increase in average maternal PM_10_ exposure during pregnancy. Our estimates, which were based on center-specific ORs estimated using a common protocol, were slightly more precise than reported by Sapkota et al. (e.g., OR = 1.03; 95% CI: 1.01, 1.05 for a 10-μg/m^3^ increase in average maternal PM_10_ exposure based on SES-adjusted estimates); but both point estimates support a comparable deleterious effect of PM_10_ on term birth weight, an indicator of fetal growth. This comparable finding supports not overrelying on statistical significance nor using it as deciding factor in the evaluation of the evidence, but rather describing the degree of confidence and precision of the estimate, which is consistent with other scientific writings on this topic. Bacchetti et al. (2005, 2008), for example, caution against inappropriate reliance on statistical significance and rather support the decision-making process evaluating the degree of confidence in the findings and the effect size. As we have shown by comparing our results to those of Sapkota et al. (2010), even though we were able to improve the signal-to-noise ratio using our approach, the interpretation of the relationship between air pollution and fetal growth remained similar.

The consistency of our findings with those of Sapkota et al. (2010), our observed consistency of combined ORs and between-center heterogeneity (τ^2^ values) for meta-analyses of ORs adjusted for center-specific covariates, and our meta-analyses of unadjusted or SES-adjusted ORs give us confidence that synthesizing effect estimates for the air pollution–pregnancy outcomes associations reported by studies with different designs is informative. Further, experience from this study could be important for policy makers in incorporating research evidence into policy—for example, by including estimates of fetal growth in the future reviews of air quality standards.

ICCAPO centers conducted unadjusted and SES-adjusted analyses according to a common analysis protocol, as well as analyses that allowed center-specific selection of covariates. In combining results across centers, there were clear trade-offs to the two approaches. Strictly specifying the models and covariates to be consistent across centers was one way to reduce methodological differences between centers. However, there might be important center-specific considerations that required inclusion of certain covariates for validity. For example, the Atlanta results relied entirely on temporal contrasts of exposure over an 11-year time period, and failing to control for long-term time trends, as was done in the unadjusted and SES-adjusted analyses, may have led to temporal confounding. This might explain why ORs adjusted for center-specific selection of covariates were different from those unadjusted or adjusted for SES only in some cases.

*Additional meta-analyses.* In their meta-analysis, Sapkota et al. (2010) estimated the association between LBW and maternal PM_10_ exposure during the first trimester (five studies) and third trimester (seven studies), but did not detect associations with exposure in either trimester (OR = 1.00; 95% CI: 0.97, 1.03, and OR = 1.00; 95% CI: 0.99, 1.01, respectively). We estimated slightly increased relative risks of term LBW in association with PM_10_ exposure during all trimesters, consistent with other previous reports (Parker and Woodruff 2008; Parker et al. 2005).

Because Cochran’s Q test of heterogeneity *p*-values for the trimester-specific analyses of PM_10_ and LBW were between 0.05 and 0.10, we reported results of both fixed-effects and random-effects meta-analyses (Higgins et al. 2002). For the second and third trimesters, there were no notable differences in estimates between the two models, whereas the random-effects OR for the association with first-trimester PM_10_ was null. Given the evidence of heterogeneity, fixed-effects model estimates for this trimester should be interpreted with caution.

Combined ORs for term birth weight as a continuous variable indicated a reduction in term birth weight associated with a 10-μg/m^3^ increase in average PM_10_ exposure during the entire pregnancy (–2.7 g; 95% CI: –7.2, 1.7 g). The association was stronger when based on ORs adjusted for maternal SES and center-specific covariates (–8.9 g; 95% CI: –13.2, –4.6 g). This may be partly explained by adjustment for gestational age at delivery by nine centers. Gestational age at delivery could confound the association between maternal exposure to air pollution and fetal growth. Although the outcome was term birth weight or LBW, there could be a 6-week difference (between 37 and 42 weeks of gestation) in gestational age at delivery. Confounding by gestational age might have had a stronger effect on the continuous birth weight analyses than on the LBW analyses.

LBW was associated with maternal PM_2.5_ exposure (combined random-effects OR = 1.10; 95% CI: 1.03, 1.18 for a 10-μg/m^3^ increase in average PM_2.5_ based on SES-adjusted ORs). The strength and direction of this association was comparable with the meta-analysis OR reported by Sapkota et al. (2010) (OR = 1.09; 95% CI: 0.90, 1.32).

*Meta-regressions.* To our best knowledge, the impact of study characteristics and exposure assessment methods on estimated associations between adverse birth weight and maternal exposure to air pollution has not been evaluated previously. Median PM_2.5_ exposure levels, PM_2.5_:PM_10_ ratios, and temporal versus spatiotemporal exposure contrasts appeared to influence the estimates of effects reported by the study centers. Parker and Woodruff (2008) suggested that variation in the composition of particulate matter may have contributed to differences in associations between maternal exposure to particulate pollution and fetal growth among seven different regions in the United States (Parker and Woodruff 2008). However, we did not have data on the composition of particulate pollutants, and therefore were not able to investigate its impact.

In our analysis, meta-regressions of the influence of temporal versus spatiotemporal exposure contrasts on associations between PM_10_ and LBW generally showed strong evidence of residual heterogeneity, in contrast to meta-regressions of median PM_2.5_ exposure levels and PM_2.5_:PM_10_ ratios (Table 4). These findings could suggest that at least part of the effect of the exposure contrast on PM_10_–term LBW associations might have been secondary to the effect of PM_2.5_:PM_10_ ratios and median PM_2.5_ levels at each center.

*Limitations.* Our meta-regressions were based on effect estimates (ORs) from 13 ICAPPO centers. As a rule of thumb, it has been suggested that 10 effect estimates are required in order to include a covariate in meta-regression (Borenstein et al. 2009). We therefore had to limit our analysis to univariate rather than multivariate meta-regressions and test the effects of each covariate separately. As a result, we could not evaluate whether our univariate results were confounded by other factors. Furthermore, other center characteristics that could have affected center-specific effect estimates, such as the prevalence of maternal smoking, were not included in our meta-regression analyses.

The ICAPPO protocol did not include data that might have been used to better characterize personal exposure, such as direct measurements of personal exposure levels (e.g., personal monitor data), pollutant levels at microenvironmental levels (e.g., indoor, outdoor, commuting) or maternal time–activity patterns. Some exposure misclassification would have resulted from the use of effect estimates based on associations with ambient levels of pollutants as a surrogate for personal exposure levels.

## Conclusion

Our combined effect estimates, which were based on effect estimates generated by 14 ICAPPO centers across the globe using a common analytical protocol, support an adverse impact of maternal exposure to particulate pollution on fetal growth. The estimated combined associations, although relatively small, could be of major public health importance considering the ubiquitous nature of particulate air pollution exposure and therefore the potential for considerable population attributable risks, particularly given evidence of both perinatal and lifelong effects of LBW on health (Balci et al. 2010; Gibson 2007). After reducing analytical differences as a possible source of heterogeneity by using a common protocol, we found that some of the heterogeneity in effect estimates by centers could be explained by differences in median PM_2.5_ exposure levels and PM_2.5_:PM_10_ ratios, suggesting geographical variation in the association between air pollution and fetal growth. In general, the direction and strength of combined estimates of association and between-center heterogeneity based on unadjusted and SES-adjusted ORs were consistent with combined estimates based on ORs adjusted for center-specific covariates. These findings highlight the contribution of study settings to inconsistencies in the available literature and can therefore increase the confidence of policy makers when summarizing existing evidence and translating it into policy.

## Supplemental Material

(344 KB) PDF
