# Supplementary material for: Maternal Exposure to Particulate Air Pollution and Term Birth Weight: A Multi-Country Evaluation of Effect and Heterogeneity
Source: Environ Health Perspect. 2013 Feb 6;121(3):267–373. doi: 10.1289/ehp.1205575 (PMC3621183; doi:10.1289/ehp.1205575)
Supplement: (344 KB) PDF [file ehp.1205575.s001.pdf]

Supplemental Material

Maternal Exposure to Particulate Air Pollution and Term Birth Weight: A Multi-Country Evaluation of Effect and Heterogeneity

Payam Dadvand,[1,2,3] Jennifer Parker,[4] Michelle L. Bell,[5] Matteo Bonzini,[6] Michael Brauer,[7] Lyndsey A. Darrow,[8] Ulrike Gehring,[9] Svetlana V. Glinianaia,[10] Nelson Gouveia,[11] Eun-hee Ha,[12] Jong Han Leem,[13] Edith H. van den Hooven,[14,15] Bin Jalaludin,[16,17,18] Bill M. Jesdale,[19] Johanna Lepeule,[20,21,22] Rachel Morello-Frosch,[19,23] Geoffrey G. Morgan,[24,25] Angela Cecilia Pesatori,[26] Frank H. Pierik,[15] Tanja Pless-Mulloli,[10] David Q. Rich,[27] Sheela Sathyanarayana,[28] Juhee Seo,[12] Rémy Slama,[21,22] Matthew Strickland,[8] Lillian Tamburic,[29] Daniel Wartenberg,[30] Mark J Nieuwenhuijsen,[1,2,3] Tracey J. Woodruff [31]

[1] Centre for Research in Environmental Epidemiology (CREAL), Barcelona, Spain

[2] Municipal Institute of Medical Research (IMIM-Hospital del Mar), Barcelona, Spain

[3] CIBER Epidemiologia y Salud Pública (CIBERESP), Spain

[4] National Center for Health Statistics, Centers for Disease Control and Prevention, Hyattsville, Maryland, USA

[5] Yale University, School of Forestry and Environmental Studies, New Haven, Connecticut, USA

[6] Department of Clinical and Experimental Medicine, University of Insubria, Varese, Italy

[7] University of British Columbia, School of Population and Public Health, Vancouver, British Columbia, Canada

[8] Department of Environmental Health, Emory University, Atlanta, Georgia, USA

[9] Institute for Risk Assessment Sciences, Utrecht University, Utrecht, the Netherlands

[10] Institute of Health & Society, Newcastle University, Newcastle upon Tyne, England, United Kingdom

[11] Department of Preventive Medicine, School of Medicine of the University of São Paulo, São Paulo, Brasil

[12] Department of Preventive Medicine, Ewha Womans University, Seoul, Republic of Korea

[13] Department of Occupational and Environmental Medicine, Inha University, Incheon, Republic of Korea

[14] Generation R Study Group, Erasmus Medical Center, Rotterdam, the Netherlands

[15] Urban Environment and Safety, TNO, Utrecht, The Netherlands

[16] Centre for Research, Evidence Management and Surveillance, Sydney, Australia

[17] South Western Sydney Local Health Districts, Sydney, Australia

[18] School of Public Health and Community Medicine, University of New South Wales, Sydney, Australia
[19] Department of Environmental Science, Policy and Management, University of California–Berkeley, Berkeley, California, USA
[20] Department of Environmental Health, Harvard School of Public Health, Boston, Massachusetts, USA
[21] Team of Environmental Epidemiology applied to Reproduction and Respiratory Health, INSERM, U823, Institut Albert Bonniot, Grenoble, France
[22] Grenoble University, U823, Institut Albert Bonniot, Grenoble, France
[23] School of Public Health, University of California–Berkeley, Berkeley, California, USA
[24] North Coast Area Health Service, Lismore, New South Wales, Australia
[25] University Centre for Rural Health–North Coast, University of Sydney, Sydney, New South Wales, Australia
[26] Department of Occupational and Environmental Health, Università di Milano, Milan, Italy
[27] Department of Public Health Sciences, University of Rochester School of Medicine and Dentistry, Rochester, New York, USA
[28] Seattle Children's Research Institute, University of Washington, Seattle, Washington, USA
[29] University of British Columbia, Centre for Health Services and Policy Research, Vancouver, British Columbia, Canada
[30] UMDNJ-Robert Wood Johnson Medical School, Piscataway, New Jersey, USA
[31] Center for Reproductive Health and the Environment. University of California–San Francisco, San Francisco, California, USA

	**Page**
Supplemental Material, Table S1	4
Supplemental Material, Figure S1	5
Supplemental Material, Figure S2	6
Supplemental Material, Figure S3	8
Supplemental Material, Figure S4	9

Supplemental Material, Table S1. Influential centers (DFBETAS value more than one) in meta-analyses.

Meta-analysis	Influential Centers
**PM_{10}-Term LBW Association**	
Unadjusted	
All studies (Random effect model)	Connecticut & Massachusetts
Adjusted for maternal socioeconomic status	
All studies (Random effects model)	California, Seoul
Trimester 1 (Fixed-effect model)	PAMPER
Trimester 1 (Random effects model)	Lombardy, PAMPER
Trimester 2 (Fixed-effect model)	-
Trimester 3 (Fixed-effect model)	-
Trimester 3 (Random effects model)	Lombardy
Adjusted for maternal socioeconomic status and center-specific covariates	
All studies (Random effects model)	California, PAMPER, Sydney
**PM_{10}-Term Birth Weight Association**	
Unadjusted	
All studies (Random effect model)	Atlanta
Adjusted for maternal socioeconomic status	
All studies (Random effects model)	Atlanta, Lombardy, Sydney
Adjusted for maternal socioeconomic status and center-specific covariates	
All studies (Random effects model)	Lombardy, Sydney
**$PM_{2.5}$-Term LBW Association**	
Unadjusted	
All studies (Random effect model)	Connecticut and Massachusetts, Seattle
Adjusted for maternal socioeconomic status	
All studies (Random effects model)	California, Connecticut and Massachusetts, Seattle
Adjusted for maternal socioeconomic status and center-specific covariates	
All studies (Random effects model)	Seattle

Supplemental Material, Figure S1. Forest plot for the random effects meta-analysis of the unadjusted (A) and adjusted[a] (B) odds ratios of term LBW in association with a 10-µg/m^3 increase in maternal PM_{10} exposure during the entire pregnancy.

A)

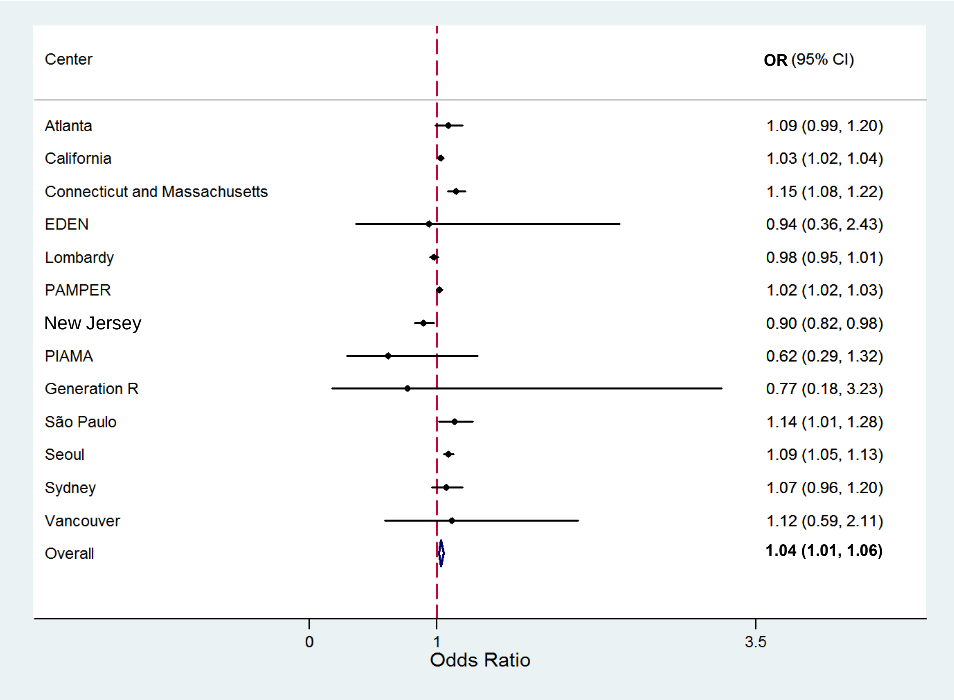

B)

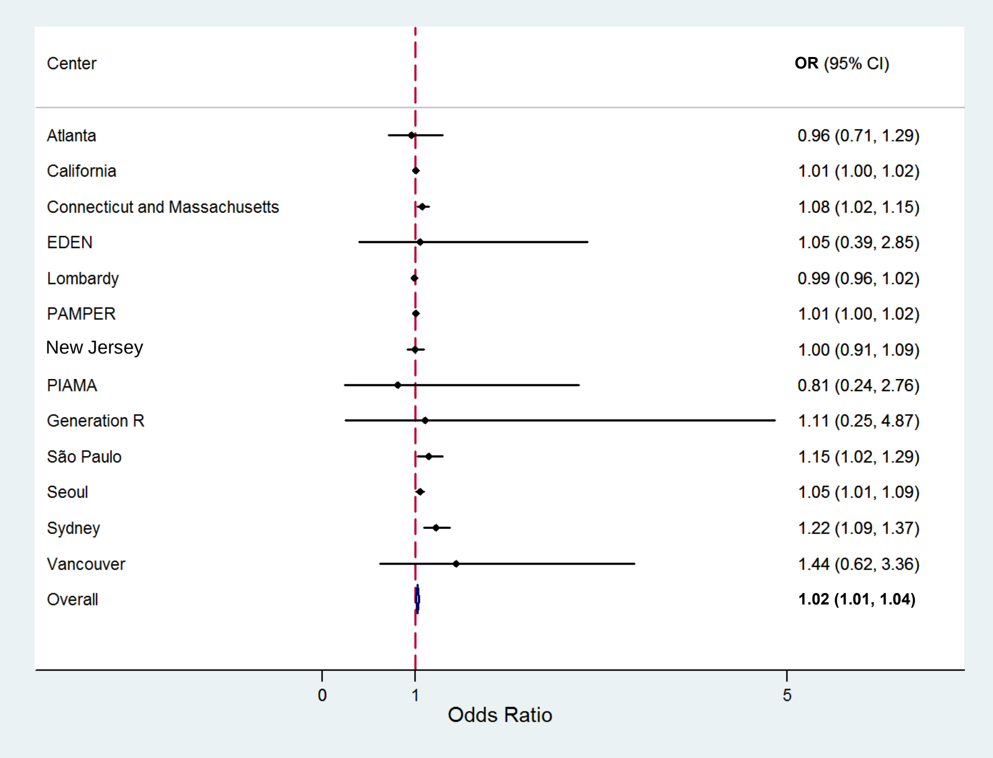

[a] Adjusted for maternal socioeconomic status and centre-specific covariates.

Supplemental Material, Figure S2. Forest plot for the fixed-effect meta-analysis of odds ratio (95% confidence interval) of term LBW in association with a 10-μg/m^3 increase in maternal PM_{10} exposure during the first (A), second (B), and third (C) trimester adjusted for maternal socioeconomic status.

A)

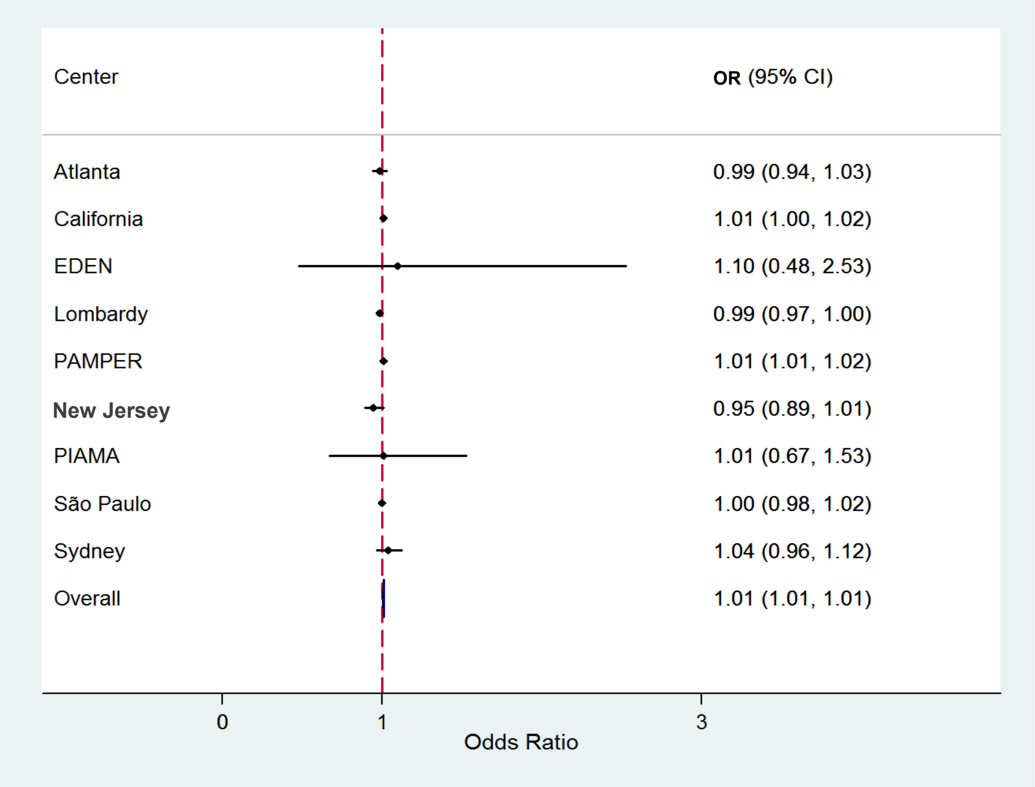

B)

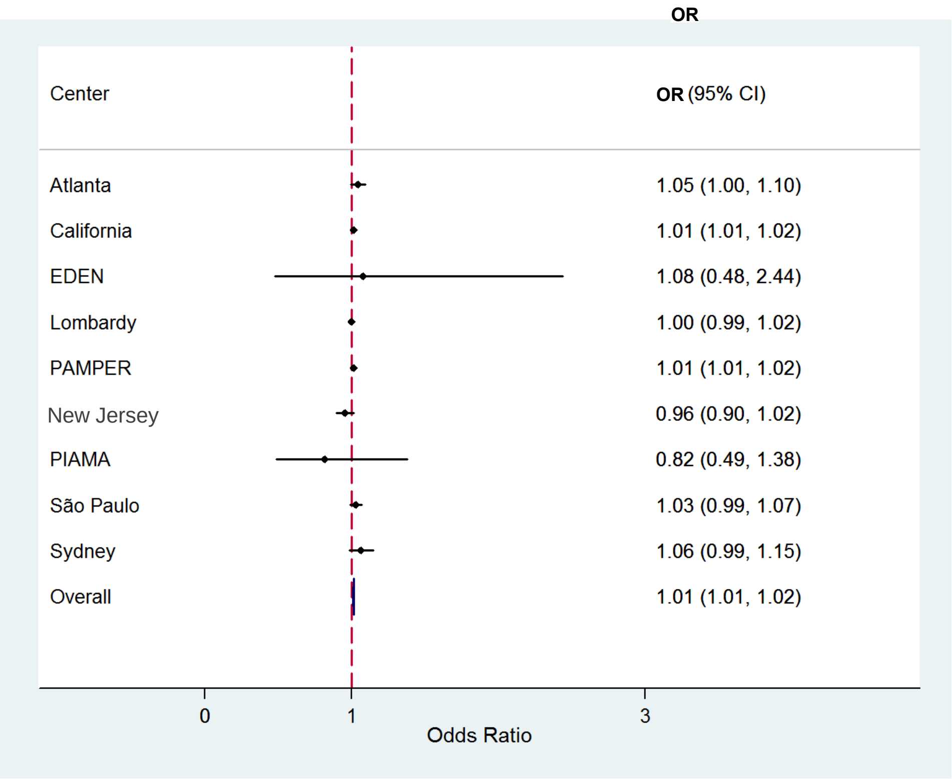

C)

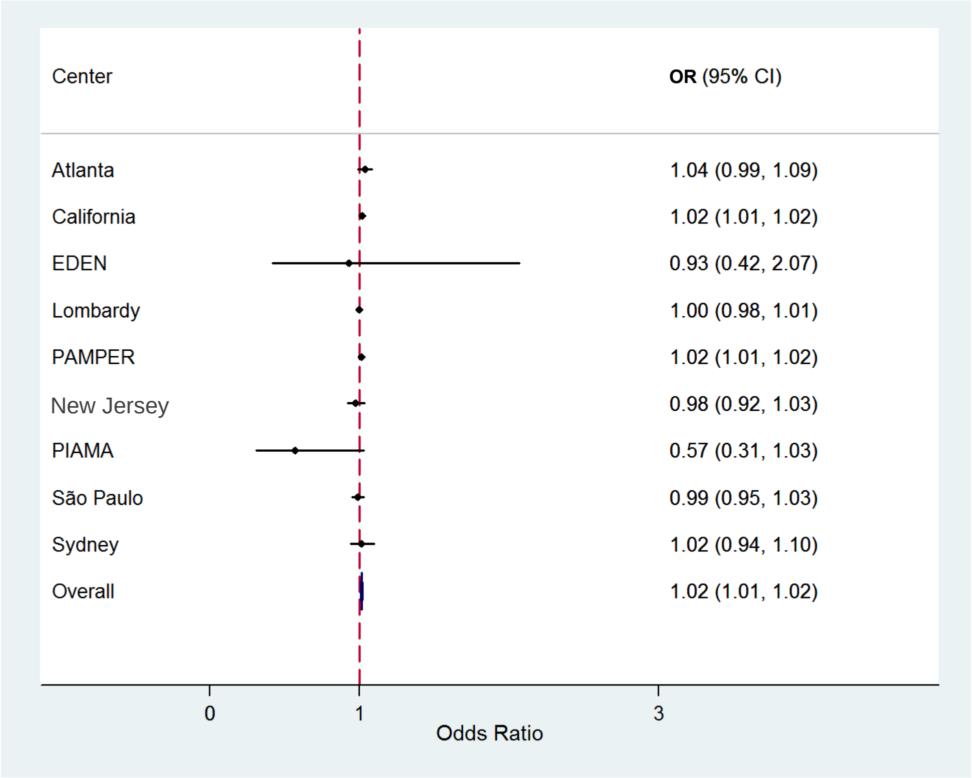

Center
OR (95% CI)
Atlanta
1.04 (0.99, 1.09)
California
1.02 (1.01, 1.02)
EDEN
0.93 (0.42, 2.07)
Lombardy
1.00 (0.98, 1.01)
PAMPER
1.02 (1.01, 1.02)
New Jersey
0.98 (0.92, 1.03)
PIAMA
0.57 (0.31, 1.03)
São Paulo
0.99 (0.95, 1.03)
Sydney
1.02 (0.94, 1.10)
Overall
1.02 (1.01, 1.02)
0
1
3
Odds Ratio

Supplemental Material, Figure S3. Forest plot for the random effects meta-analysis of the unadjusted (A) and adjusted[a] (B) change in birth weight (mean difference (95% confidence interval)) in association with a 10-µg/m^3 increase in maternal PM_{10} exposure during the entire pregnancy.

A)

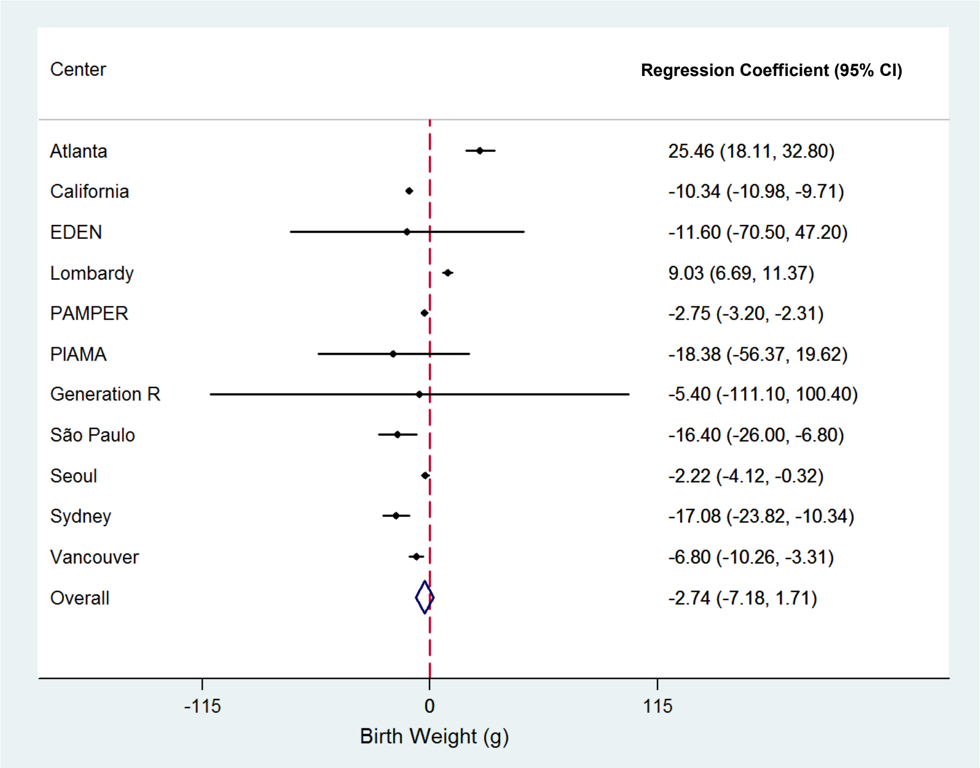

B)

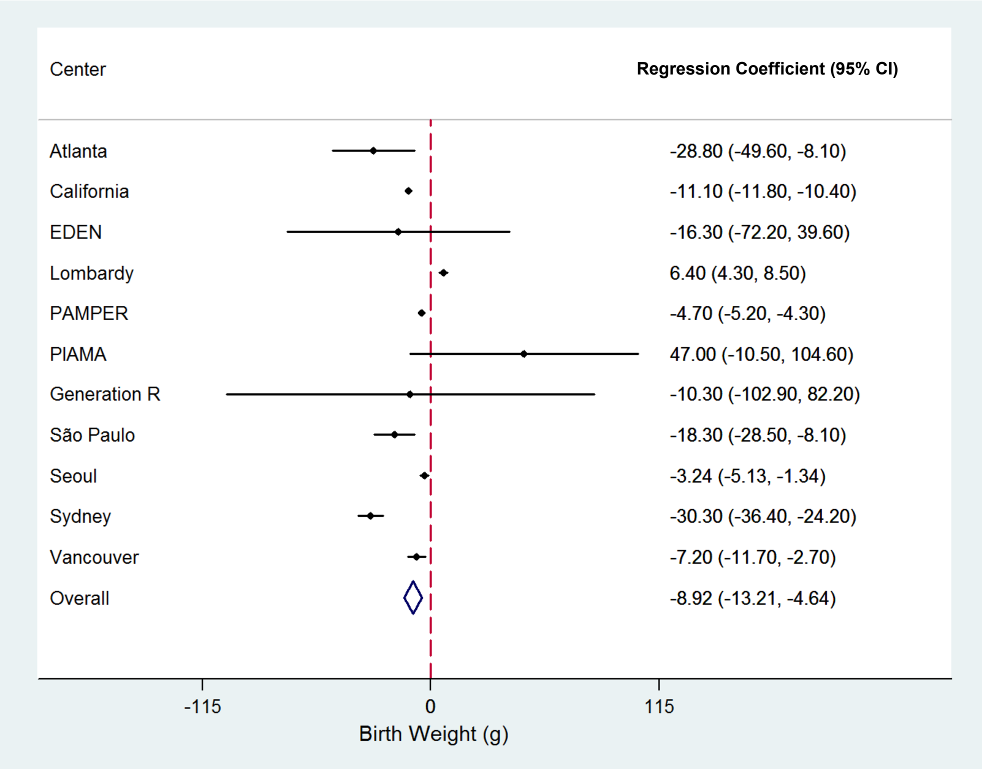

[a] Adjusted for maternal socioeconomic status and centre-specific covariates.

Supplemental Material, Figure S4. Forest plot for the random effects meta-analysis of the unadjusted (A) and adjusted[a] (B) odds ratios of term LBW in association with a 10-µg/m^3 increase in maternal $PM_{2.5}$ exposure during the entire pregnancy.

A)

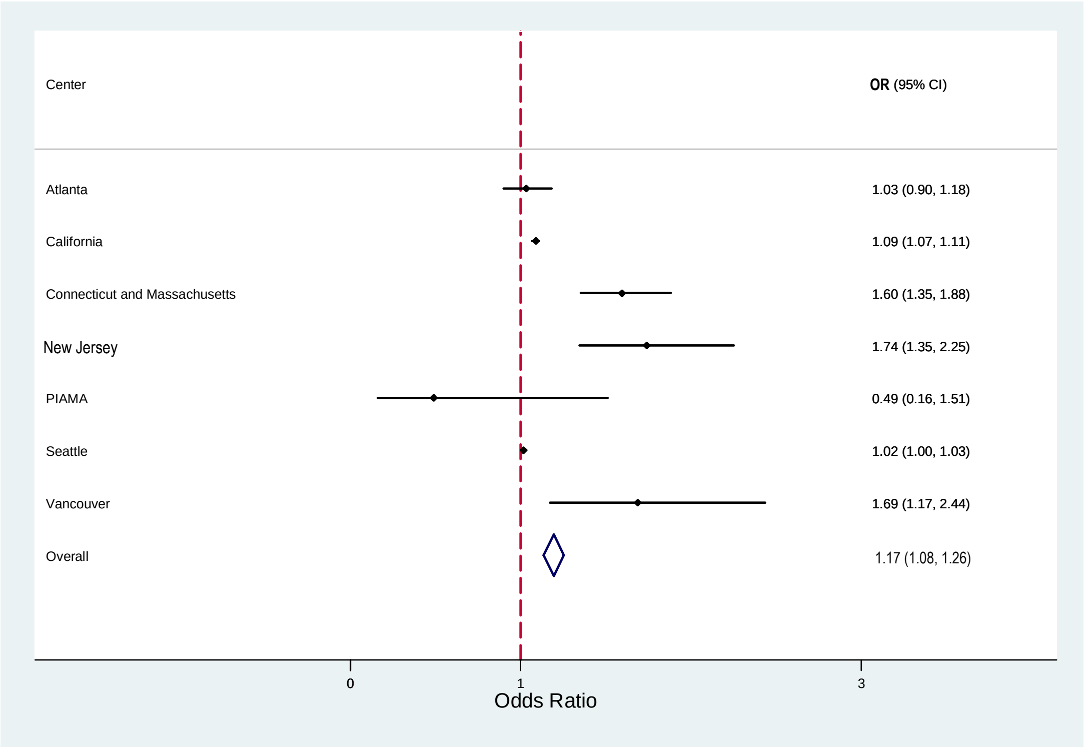

B)

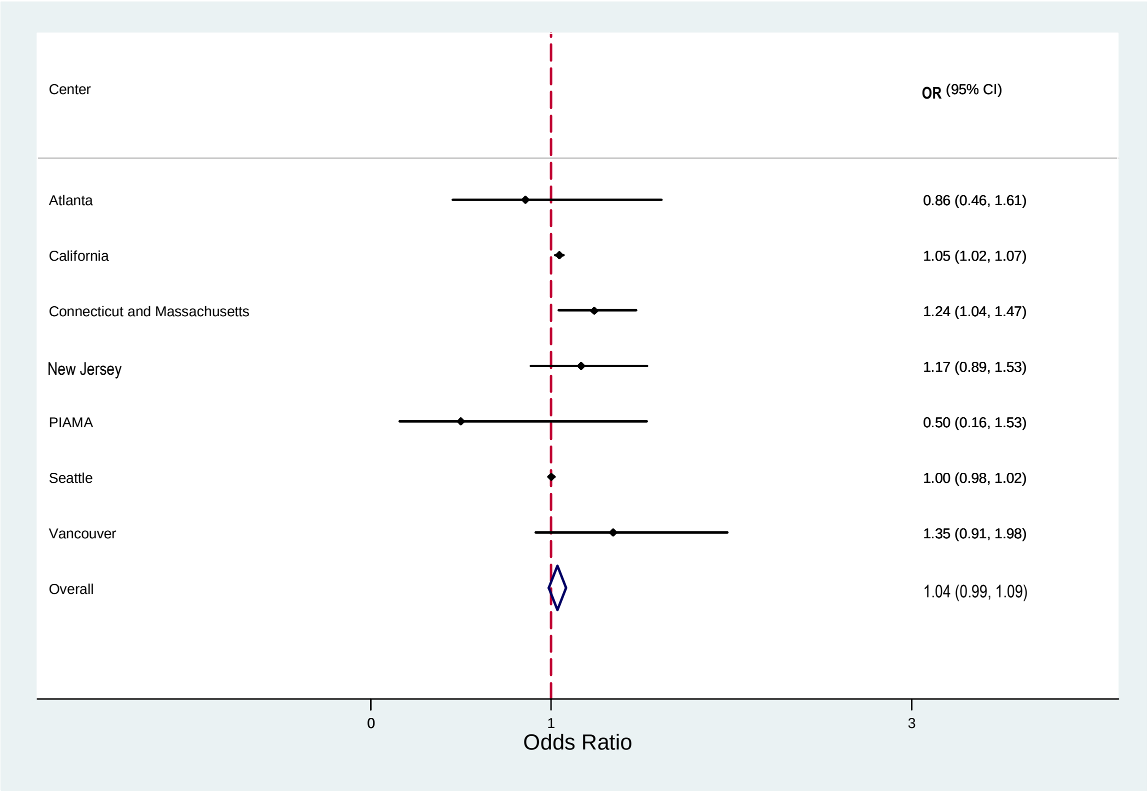

[a] Adjusted for maternal socioeconomic status and centre-specific covariates.
